# A chimeric haemagglutinin-based universal influenza virus vaccine boosts human cellular immune responses directed towards the conserved haemagglutinin stalk domain and the viral nucleoprotein

**DOI:** 10.1016/j.ebiom.2024.105153

**Published:** 2024-05-27

**Authors:** Carly M. Bliss, Raffael Nachbagauer, Chiara Mariottini, Frans Cuevas, Jodi Feser, Abdi Naficy, David I. Bernstein, Jeffrey Guptill, Emmanuel B. Walter, Francesco Berlanda-Scorza, Bruce L. Innis, Adolfo García-Sastre, Peter Palese, Florian Krammer, Lynda Coughlan

**Affiliations:** a Department of Microbiology, Icahn School of Medicine at Mount Sinai, New York, NY, USA; b Division of Cancer & Genetics and Systems Immunity University Research Institute, School of Medicine, Cardiff University, Cardiff, UK; c Center for Vaccine Innovation and Access, PATH, Seattle, WA, USA; d Department of Pediatrics, University of Cincinnati College of Medicine, Cincinnati, OH, USA; e Division of Infectious Diseases, Cincinnati Children’s Hospital Medical Center, Cincinnati, OH, USA; f Duke Early Phase Clinical Research Unit, Duke Clinical Research Institute, Durham, NC, USA; g Duke Human Vaccine Institute, Duke University School of Medicine, Durham, NC, USA; h Global Health and Emerging Pathogens Institute, Icahn School of Medicine at Mount Sinai, New York, NY, USA; i Department of Medicine, Division of Infectious Diseases, Icahn School of Medicine at Mount Sinai, New York, NY, USA; j Department of Pathology, Molecular and Cell-Based Medicine, Icahn School of Medicine at Mount Sinai, New York, NY, USA; k The Tisch Cancer Institute, Icahn School of Medicine at Mount Sinai, New York, NY, USA; l The Icahn Genomics Institute, Icahn School of Medicine at Mount Sinai, New York, NY, USA; m Center for Vaccine Research and Pandemic Preparedness (C-VaRPP), Icahn School of Medicine at Mount Sinai, New York, NY, USA; n University of Maryland School of Medicine, Department of Microbiology and Immunology, Baltimore, MD 21201, USA; o University of Maryland School of Medicine, Center for Vaccine Development and Global Health (CVD), Baltimore, MD 21201, USA

**Keywords:** Universal influenza vaccine, Haemagglutinin, Stalk, T cells

## Abstract

**Background:**

The development of a universal influenza virus vaccine, to protect against both seasonal and pandemic influenza A viruses, is a long-standing public health goal. The conserved stalk domain of haemagglutinin (HA) is a promising vaccine target. However, the stalk is immunosubdominant. As such, innovative approaches are required to elicit robust immunity against this domain. In a previously reported observer-blind, randomised placebo-controlled phase I trial (NCT03300050), immunisation regimens using chimeric HA (cHA)-based immunogens formulated as inactivated influenza vaccines (IIV) −/+ AS03 adjuvant, or live attenuated influenza vaccines (LAIV), elicited durable HA stalk-specific antibodies with broad reactivity. In this study, we sought to determine if these vaccines could also boost T cell responses against HA stalk, and nucleoprotein (NP).

**Methods:**

We measured interferon-γ (IFN-γ) responses by Enzyme-Linked ImmunoSpot (ELISpot) assay at baseline, seven days post-prime, pre-boost and seven days post-boost following heterologous prime:boost regimens of LAIV and/or adjuvanted/unadjuvanted IIV-cHA vaccines.

**Findings:**

Our findings demonstrate that immunisation with adjuvanted cHA-based IIVs boost HA stalk-specific and NP-specific T cell responses in humans. To date, it has been unclear if HA stalk-specific T cells can be boosted in humans by HA-stalk focused universal vaccines. Therefore, our study will provide valuable insights for the design of future studies to determine the precise role of HA stalk-specific T cells in broad protection.

**Interpretation:**

Considering that cHA-based vaccines also elicit stalk-specific antibodies, these data support the further clinical advancement of cHA-based universal influenza vaccine candidates.

**Funding:**

This study was funded in part by the 10.13039/100000865Bill and Melinda Gates Foundation (BMGF).

**Research in context**

**Evidence before this study**

A systematic search of PubMed/Medline was performed to evaluate studies focused on the induction of haemagglutinin (HA) stalk-specific T cells in humans following immunisation. Search terms included “influenza haemagglutinin AND stalk” or “influenza haemagglutinin AND vaccine AND cellular”, from years 2008–2024, using filters for “Clinical Trial” and “Randomized Controlled Trial”. This search yielded up to one hundred and twenty publications, including three directly related to our clinical trial (NCT03300050). All other manuscripts related to the study of HA stalk-specific antibodies, or the clinical evaluation of the therapeutic efficacy of antibodies targeting the HA stalk domain. A limited number of studies have measured T cell responses to the whole HA antigen in humans, but these did not specifically look at boosting of T cells recognising the HA stalk domain.

**Added value of this study**

In this exploratory study using cryopreserved PBMCs from a subset of participants in a clinical trial to test HA stalk focused universal vaccines based on inactivated influenza vaccine (IIV), or live attenuated influenza vaccine (LAIV) platforms, with or without use of adjuvant, we specifically evaluated boosting of HA stalk-specific T cells in humans. We showed that prime:boost immunisation regimens which included a chimeric HA (cHA), stalk-focused immunogen formulated as IIV with AS03 adjuvant was capable of boosting stalk-specific T cells in participants.

**Implications of all the available evidence**

A large body of published literature now supports a major role for antibodies recognising the HA stalk being capable of breadth of reactivity (i.e., against seasonal, pandemic and emerging avian influenza viruses), and possessing a wide range of functional and protective capacities in animal models (neutralisation, effector function activity). However, even in mice, very little is known about HA stalk-specific T cell responses, their role in protection, and which immunisation platforms or regimens may be capable of boosting these cellular effectors. Considering *(i)* the intense research efforts being conducted globally to develop optimised universal influenza virus vaccines, *(ii)* the ongoing threat that emerging pandemic viruses pose, and *(iii)* the fact that improving our understanding of correlates of protection for influenza vaccines is a strategic priority for funding agencies (e.g., NIAID), our findings will be useful in informing future clinical trial design.

## Introduction

Influenza A viruses (IAV) are responsible for annual epidemics resulting in ∼290,000–650,000 global deaths per year.^1^ In addition to seasonal epidemics, IAVs can cause sporadic pandemics. The potential for emergence of novel reassortant viruses from the animal reservoir which have pandemic potential, represents an ongoing concern, particularly the pan-zootic clade 2.3.4.4b H5N1 avian influenza viruses.^2^^,^^3^

The variable effectiveness of seasonal influenza virus vaccines is well-documented.^4^, ^5^, ^6^ Conventional inactivated influenza vaccines (IIVs) elicit largely strain-specific immune responses which are directed towards the globular head domain of the main surface glycoprotein, the haemagglutinin (HA), with limited induction of responses towards the HA stalk domain. The HA head is immunodominant, and when vaccine strains are well-matched to circulating influenza viruses, antibodies (Abs) recognising the HA head can confer protection from disease. Unfortunately, the HA head is antigenically variable and tolerates the accumulation of mutations which can result in virus escape from protective Abs elicited by licensed seasonal influenza vaccines.^7^ Conventional unadjuvanted seasonal IIVs are limited in the induction of cellular immune responses, and largely induce humoral immunity.^8^ Although live attenuated influenza vaccines (LAIV) do elicit T cell responses, these are preferentially recommended for use in children due to improved efficacy in this age group as compared with adults.^9^^,^^10^ Seasonal vaccines also lack suitability for pandemic preparedness for several reasons. First, IAVs are zoonotic viruses which circulate in a broad range of animal species, and a diverse number of distinct HA subtypes exist (H1–H16 and H19,^11^ in addition to bat HAs H17 and H18).^12^ IAV HAs are sub-divided phylogenetically into group 1 (G1) and group 2 (G2). The strain-specificity of conventional influenza virus vaccines means that they would confer little or no protection against emerging viruses with HA subtypes which do not normally circulate in humans (e.g., avian H5). Therefore, it is clear that efforts to develop and evaluate novel vaccine platforms and strategies capable of eliciting increased breadth of reactivity against multiple IAVs are urgently needed.

Universal influenza virus vaccines are in various stages of pre-clinical and clinical development. Development strategies centre on re-directing immune responses away from antigenically variable epitopes in the HA head, and re-focusing immunity towards highly conserved viral antigens or domains.^13^, ^14^, ^15^, ^16^, ^17^, ^18^, ^19^, ^20^, ^21^ One such target is the conserved stalk (or stem) domain of HA, which plays an important role in mediating viral fusion and entry.^12^ Unlike the antigenically variable HA head domain, the stalk is highly conserved, and is limited in its tolerance of mutations without compromising viral fitness,^22^, ^23^, ^24^ although this may vary between the stalks of G1 and G2 HAs.^25^ Nonetheless, there is extensive evidence that Abs directed towards the HA stalk can confer protection in animal models using a diverse range of functional activities.^26^, ^27^, ^28^, ^29^, ^30^, ^31^, ^32^ Importantly, HA stalk Abs have recently been identified as a potential correlate of protection in human cohort studies of natural influenza virus infection.^33^^,^^34^ Collectively, the high degree of conservation within the stalks of G1, and G2 IAVs, as well as documented evidence for stalk-specific immunity conferring protection *in vivo*, make the stalk an attractive target for next-generation influenza virus vaccines.

Strategies to maximise immune recognition of the HA stalk have included the use of viral vectored vaccines,^35^ mRNA,^27^ or nanoparticle-based platforms,^32^ the inclusion of adjuvants to increase breadth of immunity, as well as the design of structurally stabilised headless HA immunogens,^29^^,^^36^, ^37^, ^38^ or chimeric HA (cHA) immunogens.^26^^,^^28^^,^^39^, ^40^, ^41^ The latter approach involves grafting the head domain of a HA subtype which is exotic to humans (e.g., H8), onto the stalk domain of a HA subtype which circulates in humans (e.g., H1) to produce a chimeric antigen (e.g., cH8/1). Sequential immunisation with distinct cHA-based immunogens in which the stalk domain remains the same upon each immunisation, but the HA head domain is swapped out (e.g., cH8/1 followed by cH5/1), leads to re-focusing of humoral immune responses away from the immunodominant head and towards the immunosubdominant HA stalk. Importantly, unlike headless HAs, the cHA design is compatible with conventional influenza vaccine production, including LAIV and IIV platforms.^13^^,^^42^ Sequential cHA immunisation regimens using cHA platforms have been successful in animal models, demonstrating that heterosubtypic protection can be achieved against a diverse range of influenza viruses.^40^ Therefore, a vaccine capable of eliciting breadth of reactivity against diverse G1 or G2 HAs, such as H1, H2, H5, or H3, H7 and H10,^43^ would represent an advance over conventional seasonal influenza virus vaccines and would be ideally suited to pandemic preparedness and stockpiling.^44^^,^^45^

We previously reported the safety and humoral immunogenicity of G1 cHA-based LAIV and IIV vaccine candidates in humans (NCT03300050).^13^^,^^15^^,^^44^^,^^46^ We confirmed that the cHA immunisation approach successfully boosted cross-reactive stalk Abs in humans, which were sustained for up to 18 months post-immunisation (end-point for analysis).^13^ These Abs exhibited breadth of reactivity against diverse G1 HAs,^44^ and targeted the central stalk epitope (i.e., CR9114),^47^^,^^48^ as well as a membrane-proximal, broadly neutralising anchor epitope.^48^^,^^49^ Furthermore, stalk Abs elicited by this vaccination regimen displayed a range of functions, including virus neutralisation, as well Fc-mediated antibody-dependent cellular cytotoxicity (ADCC) and antibody-dependent cellular phagocytosis (ADCP), mechanisms which have been identified as contributing to heterosubtypic protection *in vivo* in animal models.^7^^,^^31^

The purpose of the current study was to conduct tertiary exploratory analyses to evaluate T cell responses in humans following immunisation with the cHA LAIV and/or adjuvanted/non-adjuvanted cHA IIV platforms in the previously reported clinical trial (NCT03300050). The majority of previous universal vaccine studies have focused on internal viral antigens, such as the nucleoprotein (NP) or the matrix protein-1 (M1), which have been reported to be dominant targets for cross-reactive cellular immune responses following influenza virus infection.^50^, ^51^, ^52^, ^53^ Similar to the HA stalk domain, NP and M1 are highly conserved, and cross-reactivity of NP-specific T cells against heterosubtypic viruses has been reported in humans.^54^ Importantly, T cells recognizing internal viral antigens or influenza-specific T cells have also been identified as a potential correlate of protection in longitudinal cohort studies,^55^^,^^56^ and in human challenge experiments.^57^^,^^58^ However, in contrast, little work has been done to investigate the role of HA stalk-specific T cell responses, despite the importance of the HA stalk in universal influenza virus vaccine development.

In this study, we report that H1 stalk-specific T cells in humans are successfully boosted following intramuscular (*i.m.*) immunisation with adjuvanted cHA-based universal influenza virus vaccine candidates. These data complement prior clinical analyses, which clearly demonstrated that adjuvanted cHA-IIV formulations increase cross-subtype immunity, and elicit HA stalk-specific Ab responses which display a range of functional activities associated with protection.^13^^,^^14^^,^^44^^,^^46^ A vaccine which simultaneously elicits broadly cross-reactive humoral *and* cellular immune responses, represents an ideal universal influenza virus vaccine candidate.

## Methods

### Objectives

The original clinical study was designed to evaluate the safety and immunogenicity of a prime:boost regimen comparing intranasal (*i.n*) LAIV, or intramuscular (*i.m*), AS03-adjuvanted split inactivated influenza vaccine (IIV) prime, followed by an *i.m* boost with IIV, administered with or without AS03 adjuvant. The primary outcomes of the trial (registered with ClinicalTrials.gov, NCT03300050) have been reported previously and the clinical study protocol is available at https://clinicaltrials.gov/study/NCT03300050.^13^^,^^14^ The selection of AS03 in G4 (IIV8/AS03-IIV5/AS03) was to act as a bridging group to a parallel GlaxoSmithKline (GSK) first-in-human clinical trial (NCT03275389) initiated prior to this study.^15^ Separate clinical studies with influenza vaccines had previously shown that AS03 could facilitate antigen sparing, and could enhance both humoral and cellular immune responses.^59^, ^60^, ^61^, ^62^, ^63^ This manuscript reports the findings of exploratory immunological analyses to measure T cell responses to HA stalk and NP in peripheral blood mononuclear cells (PBMCs).

### Ethics

The Cincinnati Children’s Hospital Medical Center (CCHMC) Institutional Review Board (IRB) served as the central IRB for review, approval and overview of this trial, as previously described (Protocol #2017-4461).^13^^,^^14^ Written, informed consent was obtained from all participants. Descriptions of planned T cell analysis by ELISpot are detailed in section 7.3.2, 7.3.2.3, Table 12 and Table 13 in the published study protocol, available at https://clinicaltrials.gov/study/NCT03300050.

### Vaccines

The trial evaluated prime:boost regimens of an LAIV and two IIVs, as previously described.^13^^,^^14^ Briefly, the LAIV was manufactured in embryonated chicken eggs by Meridian Life Sciences in Memphis, Tennessee and formulated in sterile saline. It consisted of a chimeric H8/1 HA (head domain from A/mallard/Sweden/24/02 [H8N4], stalk domain from A/California/04/09 [H1N1]), an N1 neuraminidase (NA) from A/California/04/09, and the internal genes of the A/Leningrad/134/17/57 virus.^13^^,^^64^, ^65^, ^66^ The LAIV was administered *i.n* at a 10^7.5^ 50% egg infectious dose. The first IIV carried an identical cH8/1 HA to the LAIV, and a second IIV carried a chimeric H5/1 HA (head domain from A/Vietnam/1203/04 [H5N1], stalk domain from A/California/04/09 [H1N1]). Both were rescued with the same A/PR/8/34 (H1N1) backbone and manufactured in embryonated chicken eggs by GSK (Wavre, Belgium), as described previously.^13^^,^^14^ Split-virion IIVs were administered *i.m* in a volume of 0.5 mL, with either phosphate buffered saline (PBS) or AS03 adjuvant. The antigen content with IIVs was 15 μg of HA (cH5/1 or cH8/1). Corresponding control groups received saline *i.n* or PBS *i.m*.

### Study design

The trial consisted of 3 vaccination groups and 2 placebo groups, outlined in Table 1. Full methods for this randomised, placebo-controlled, observer-blind phase I clinical vaccine trial, along with detailed inclusion and exclusion criteria, have been previously described and can be found at https://clinicaltrials.gov/study/NCT03300050.^13^^,^^14^ Briefly, 65 volunteers were recruited across 2 study sites: CCHMC (Cincinnati, OH, USA); and Duke University Early Phase Clinical Research Unit (Durham, NC, USA). Volunteers were block-randomised by site in a ratio of 4:3:1:3:2 to receive either LAIV8-IIV5/AS03, LAIV8-IIV5, SALINE-PBS, IIV8/AS03-IIV5/AS03, or PBS-PBS. Group 1 (G1) received cH8/1N1 LAIV *i.n* on day 1 followed by AS03-adjuvanted cH5/1N1 IIV *i.m* on day 85 (denoted G1: LAIV8-IIV5/AS03). Group 2 (G2) received a similar vaccination regimen but with a non-adjuvanted booster vaccination (denoted G2: LAIV8-IIV5). Group 3 (G3) was the placebo control group for G1 and G2, receiving normal saline *i.n* on day 1 and PBS *i.m* on day 85 (denoted PLACEBO). Priming vaccinations for G1-3 were carried out in a containment unit, whereby participants were required to stay for at least five days post-vaccination, or until they were confirmed to be virus-negative by real-time PCR of oropharyngeal and nasal swabs on three consecutive days (cut-off was a cycle threshold value of ≤40). Group 4 (G4) received AS03-adjuvanted cH8/1N1 IIV *i.m* on day 1 followed by AS03-adjuvanted cH5/1N1 IIV *i.m* on day 85 (denoted G4: IIV8/AS03-IIV5/AS03). Group 5 (G5) was the placebo for G4, receiving PBS *i.m* on day 1 and day 85 (denoted PLACEBO).

**Table 1:** Clinical trial vaccine groups.

Study groups	Subjects enrolled	Included in analysis	Dose 1 (Prime, D1)		Dose 2 (Boost, D85)		Exclusions from analysis (timepoint: reason)
			Treatment	Route	Treatment	Route	
G1	*n = 20*	*n = 10*	cH8/1N1 LAIV	*i.n*	cH5/1N1 IIV + AS03_A_	*i.m*	V11: insufficient cells V11: insufficient cells (NP)
G2	*n = 15*	*n = 10*	cH8/1N1 LAIV	*i.n*	cH5/1N1 IIV	*i.m*	V11: insufficient cells (NP)
G3	*n = 5*	*n = 2*	Normal saline	*i.n*	PBS	*i.m*	No exclusions
G4	*n = 15*	*n = 10*	cH8/1N1 IIV + AS03_A_	*i.m*	cH5/1N1 IIV + AS03_A_	*i.m*	No exclusions
G5	*n = 10*	*n = 8*	PBS	*i.m*	PBS	*i.m*	V11: insufficient cells (NP) V12: plate fail, high DMSO V12: poor cell viability

A total of *n = 10* subjects from the three vaccine groups (G1, 2 and 4) were allocated for T cell analysis, and an *n = 2 and n = 8* respectively from each placebo group (G3 and G5, denoted G3+5: PLACEBO) were combined for analysis. In some cases, on the day of assay performance, insufficient cell quantities were recovered after cryopreservation, or cell viability was low. As a result, at some specific timepoints, selected peptide stimulations or participant samples were omitted from the assay or from analyses for selected wells. Information and justification for sample exclusion from analysis is provided in Table 1. Operators were blinded to the treatment groups until laboratory and data analysis were completed.

### Sample size and PBMC pick list

ELISpot assay operators were blinded to clinical groupings until locking of the T cell analytical database. To enable an equal subset of volunteers from across all groups to be evaluated for cellular immune responses, a pick list was generated by staff at The Emmes Company, LLC, providing 10 volunteers per group across G1, G2 and G4, and 10 volunteers split across PLACEBO volunteers in G3 (*n = 2*) and 5 (*n = 8*). The pick list was selected randomly from participants that completed both prime and boost immunisation interventions.^13^^,^^14^ This sampling represented 62.5–100% of the participants in the original trial: 62.5% for G1 (*n = 10* out of 16), 76.9% for G2 (*n = 10* out of 13), 66.7% for G4 (*n = 10* out of 15), 100% for PLACEBO G3 (*n = 2* out of 2) and 80% for G5 (*n = 8* out of 10). Selection was also dependent on the availability of sufficient numbers of cryopreserved PBMC vials at D1, D8, D85 and D92 for the same volunteer, to allow tracking of T cell responses at baseline (D1), 7 days post-prime (D8), pre-boost (D85) and at 7 days post-boost (D92). Importantly, all participants in this subset analysis received their prime immunisation in December 2017, thereby eliminating confounding factors related to time trends. Furthermore, none of the participants in the T cell analysis tested positive for IAV infection during our analysis window (D1–D92). As the T cell assays were tertiary exploratory assays performed once other priority assays had been completed, our sample size (*n = 10*/group) was largely defined by the availability of cryopreserved PBMCs. Demographics for the participants selected for T cell analysis are shown in Table 2.

**Table 2:** T cell analysis participant characteristics by vaccine group.

	Total *n = 40*	G1: LAIV8-IIV5/AS03 *n = 10*	G2: LAIV8-IIV5 *n = 10*	G4: IIV8/AS03-IIV5/AS03 *n = 10*	G3/5: PLACEBO *n = 2/n = 8*
**Age (years)**					
Median (IQR)	28 (25.00–31.75)	29.5 (24.25–31.50)	27 (23.50–30.25)	28 (26.25–35.50)	28.5 (25.00–32.50)
**Sex**					
Female	25 (62.5%)	8 (80%)	6 (60%)	6 (60%)	5 (50%)
Male					
**Ethnicity (self-reported)**					
Not hispanic or Latino		10 (100%)	8 (80%)	10 (100%)	10 (100%)
Hispanic or Latino		0	2 (20%)	0	0
Not reported		0	0	0	0
Unknown		0	0	0	0
**Race (self-reported)**					
Black or African American		7 (70%)	6 (60%)	9 (90%)	7 (70%)
White		3 (30%)	2 (20%)	1 (10%)	3 (30%)
Multiple		0	1 (10%)	0	0
Unknown		0	1 (10%)	0	0

### PBMC isolation

Blood samples for PBMC analysis were taken pre-vaccination (denoted D1), 7 days post-prime vaccination (denoted D8), 84 days post-prime but pre-boost vaccination (denoted D85), and 7 days post boost vaccination (denoted D92). PBMCs were isolated at the study sites, CCHMC and Duke, and cryopreserved samples provided for T cell analyses.

### Peptide preparation

Protein sequences for A/Michigan/45/2015 H1 HA stalk domain and NP were split *in silico* into 15mer, 19mer or 20mer peptide sequences overlapping by 10 amino acids (Supplementary Tables S1 and S2, respectively). Additional peptides were synthesised to act as positive controls. For the latter purpose we used an adaptation of the gold-standard “CEF” peptide pool,^67^ consisting of known CD8^+^ T cell epitopes from cytomegalovirus (CMV = “C”) and Epstein–Barr virus (EBV = “E”), but without influenza (flu = “F”) virus peptides (Supplementary Table S3).^67^^,^^68^ AbClonal (MA, USA) synthesised the peptides to 70% purity with free amino and carboxyl acid groups. Lyophilised peptides were stored at −20 °C until reconstitution and pooling. For reconstitution, peptides were warmed to room temperature for at least 1 h, then centrifuged at 500 g for 1 min, before adding dimethyl sulfoxide (DMSO) to result in a specified final peptide concentration of 25–100 mg/mL. Once reconstituted, peptides were vortexed then pooled according to antigen in culture media, consisting of Roswell Park Memorial Institute (RPMI) media supplemented with 10% (v/v) foetal bovine serum (FBS), 100 U/mL penicillin, 100 μg/mL streptomycin and 2 mM L-glutamine (complete RPMI is denoted R10). The HA stalk peptides were split across 4 separate pools (P1-4) and NP across 5 separate pools (P1-5). Each pool stock contained 20 μg/mL of each peptide, and had 6–10 peptides per pool (Supplementary Tables S1 and S2). Peptide pools were aliquoted according to the Enzyme-Linked ImmunoSpot (ELISpot) plate layout into 96 well plates. Negative control wells consisted of R10 plus an equivalent volume of DMSO as was added to the peptide wells. Peptide plates were sealed and stored at −80 °C until use. Peptide plates were freeze-thawed a maximum of 3 times, with short term storage at −20 °C. Positive control phytohaemagglutinin-L (PHA-L; Vector Laboratories, CA, USA; #L-1110) was reconstituted in PBS as per the manufacturer’s instructions, diluted in R10 to 20 μg/mL, aliquoted into single use vials and stored at −20 °C until use.

### PBMC thawing and counting

Cryovials containing frozen PBMCs were removed from liquid nitrogen storage and partially thawed in a 37 °C water bath, and then added to R10 with 25 U/mL benzonase (MilliporeSigma, MO, USA; #70664-3). Samples were counted manually using a glass haemocytometer. Live and dead cell counts were measured, recording 2 counts per technical replicate of each sample. To maintain accuracy, samples were re-suspended in a smaller volume and re-diluted to obtain a more accurate count if the mean count was less than 30 cells. Counted PBMC samples were re-suspended at 4 × 10^6^ PBMC/mL and stored in a humidified incubator at 37 °C with 5% CO_2_ until ELISpot assay plating. Counting operator was consistent across all samples tested.

### ELISpot assay

*Ex vivo* ELISpot assays were performed using Multiscreen-IP filter plates (MilliporeSigma, MO, USA; #MAIPS4510), human interferon gamma (IFN)-γ Flex streptavidin alkaline phosphatase (SA-ALP) antibody kits (Mabtech, OH, USA: #3420-2A), and 5-Bromo-4-chloro-3-indolyl phosphate with nitro blue tetrazolium (BCIP NBT)-plus chromogenic substrate (Europa Bioproducts, Ipswich UK: #NBTH-1000). IFN-γ capture antibody (1-D1K) was diluted to 10 μg/mL in carbonate bicarbonate buffer, then 50 μL/well coated onto ELISpot plates. Plates were wrapped to avoid evaporation, and stored for up to 7 days at 4 °C. Plates were then blocked with 100 μL R10 culture media, and kept at room temperature for 2–8 h before plating. Blocking media was removed, and 50 μL of the 20 μg/mL thawed peptide suspension added per well. R10 with a corresponding concentration of DMSO was added to negative control wells, and 20 μg/mL PHA-L added to the positive control wells. PBMC samples at 4 × 10^6^ PBMC/mL were mixed, then 50 μL added per well, to give final stimulant concentrations of 10 μg/mL with 2 × 10^5^ PBMC per well. Plates were placed in a humidified incubator at 37 °C with 5% CO_2_. After 18–20 h of incubation, plates were washed 6 times using PBS 1% (v/v) Tween-20. Secondary antibody (7-B6-1-Biotin, Mabtech) was diluted to 1 μg/mL, and 50 μL added per well. Plates were incubated for 2–4 h at room temperature. Secondary antibody was removed, plates washed 6 times using PBS 1% (v/v) Tween-20. Streptavidin alkaline phosphatase was diluted 1:1000, and added 50 μL/well for 1–2 h at room temperature. An aliquot of BCIP/NBT Plus developer was warmed to room temperature. Plates were washed 6 times using PBS 1% (v/v) Tween-20, then 50 μL developer added per well for 3 min. Development was halted by washing the plate with tap water. Plates were shielded from light and left to dry overnight, before wrapping in foil until automated plate counting.

### Automated plate counting

Plates were counted using an ImmunoSpot S5 Analyzer (ImmunoSpot, Shaker Heights, OH) in the Human Immune Monitoring Core at ISMMS using SmartCount settings, with identical read and count settings for all plates. Immunospot quality control (QC) settings were used to check individual wells and adjust counts to remove artifacts (e.g., fibre removal). An annotation key was inserted into any adjusted wells, raw data exported to Excel, and an image of the plate printed and cross-checked against raw data.

### ELISpot quality control (QC)

Spot counts were averaged across triplicate wells, or duplicate wells if PBMC numbers were limited. Outlier values were excluded from triplicate data if a >3-fold difference from other 2 values was recorded. One such outlier was excluded across the entire dataset. Average counts were multiplied by five, to give a spot forming unit (SFU) response per million PBMC (SFU/10^6^ PBMCs). Plate pass/fail criteria were pre-defined by positive/negative QC: with the cut-off for PHA-L positive control ELISpot responses set at >800 SFU/10^6^ (or blackout wells), and the DMSO/R10 negative control wells having less than 125 SFU/10^6^ PBMC. Over 98.7% of samples passed full negative/positive ELISpot plate QC, with only one volunteer ELISpot (V12) excluded due to high DMSO background, and one volunteer ELISpot (V12) excluded due to poor cell viability (and subsequently, plate failure due to no response in PHA-L positive control wells). All remaining samples passed QC, with blackout confirmed for all PHA-L wells, and negative control wells exhibiting a median of 8 SFU/10^6^ PBMCs (well below the pre-defined cut-off threshold).

Autologous responses from DMSO/R10 negative control wells were background subtracted from each individual peptide pool to give the ELISpot response. ELISpot responses to individual peptide pools (HA stalk or NP) following background subtraction were considered positive if ≥ 18 SFU/10^6^ PBMCs were detected. This value was defined as the median plus 2MAD (median absolute difference) of individual negative control wells across the entire QC’d dataset, with values above this considered to reach the positive threshold (PT). For whole antigen analyses, responses to individual pools were summed for the HA stalk and NP antigens, and similarly, PTs for summed pools were determined by multiplying up the PT value for DMSO/R10, resulting in cut-offs for positivity of 73 SFU/10^6^ PBMCs for HA stalk (x4 pools), and 92 SFU/10^6^ PBMCs for NP (x5 pools). PT cut-offs are indicated on each figure as a dashed line.

### Statistics

**Fig. 1:**
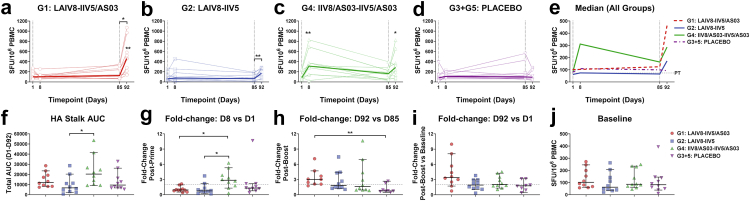
**HA stalk-specific T cell responses following immunisation with cHA-based universal influenza virus vaccine candidates.** Cryopreserved human PBMCs obtained prior to immunisation (D1), seven days post-prime (D8), pre-boost (D85) and seven days post-boost (D92) were stimulated with pools of overlapping peptides corresponding to the H1 stalk of A/Michigan/45/2015, and IFN-γ secretion measured by ELISpot. **(a–d)** Time course of individual HA stalk-specific T cell responses presented as SFU/10^6^ PBMCs for each volunteer (*n = 10/group*, single biological replicate from duplicate/triplicate wells). The median is shown as a heavy line. Statistical analyses on intra-group paired data (i.e., different timepoints for each individual volunteer) were performed using the non-parametric Friedman test with Dunn’s correction for multiple comparisons. Volunteers with missing timepoints were excluded from the Friedman analysis, but due to the limited sample size, all volunteer responses are represented graphically. Exclusions due to isolated missing timepoints are outlined in Table 1. Statistical significance icons are shown on the graph as ∗*p < 0.05*, ∗∗*p < 0.01*. Icons denote statistically significant differences between **(a)** D92 and D1 (∗∗*p = 0.0022*) and D92 versus D85 (∗*p = 0.019*) for G1 (LAIV8-IIV5/AS03), **(b)** D92 versus D85 (∗∗*p = 0.0016*) for G2 (LAIV8-IIV5) and **(c)** D8 versus D1 (∗∗*p = 0.0055*) and D92 versus D1 (∗*p = 0.036*) for G4 (IIV8/AS03-IIV5/AS03). **(e)** Median HA stalk-specific IFN-γ ELISpot response for each group. Dashed vertical grey line indicates prime (D1) and boost (D85) immunisation timepoints, and horizontal dashed grey line indicates the positive threshold (PT) for summed HA-stalk responses, which represents the median+2X median absolute deviation (MAD) for DMSO control wells corrected for summed pools (PT = 73 SFU/10^6^ PBMCs). **(f)** Total area under the curve (AUC) for HA stalk-specific IFN-γ ELISpot response from D1–D92. Inter-group comparisons were analysed using the Kruskal–Wallis test with Dunn’s correction for multiple comparisons (∗*p = 0.033*). **(g)** Fold-change in individual IFN-γ responses post-prime, D8 versus D1, for each vaccine group. Inter-group comparisons were analysed using the Kruskal–Wallis test with Dunn’s correction for multiple comparisons, with G4 response fold change increased as compared with G1 (∗*p = 0.034*) and G2 (∗*p = 0.018*). **(h)** Fold-change in individual IFN-γ responses post-boost (D92 versus D85) for each vaccine group. Inter-group comparisons were analysed using the Kruskal–Wallis test with Dunn’s correction for multiple comparisons (∗∗*p = 0.0081*). **(i)** Fold-change in individual IFN-γ responses at D92 versus D1 for each vaccine group. **(j)** Baseline responses to HA stalk peptides in each vaccine group. Solid line denotes median with 95% confidence intervals (CI) for **f–j**. Dashed grey line indicates ≥2-fold elevated T cell responses after vaccination for **g–i**.

For all analyses, control G3 and G5 were pooled and designated as PLACEBO. All data were evaluated for skewness and normal distribution, and the appropriate tests applied. For comparisons of background-subtracted ELISpot responses in the same volunteer at different timepoints (intra-group comparisons), the non-parametric Friedman test was used with Dunn’s correction for multiple comparisons for D1 versus D8, D1 versus D92 and D85 versus D92. Volunteers with missing data points were omitted from the Friedman analysis (see Table 1 for exclusions), but all data points are shown on the graph for HA stalk and NP stalk responses. For simplicity the median ELISpot response representing all data is shown for each group (Fig. 1, Fig. 3e). Area under the curve (AUC) analyses were used to capture total responses over time. When timepoints were missing due to insufficient cells, or ELISpot plate failures, the mean of the group at that timepoint was used to impute the value, as previously described^17^ (see Table 1 for exclusions). Fold-change was calculated through dividing the ELISpot response (in SFU/10^6^ PBMCs) at a given timepoint by that of a previous timepoint, on a volunteer-by-volunteer basis. For inter-group comparisons of AUC or fold-change, the non-parametric Kruskal–Wallis test with Dunn’s correction for multiple comparisons was used to determine differences between the median AUC or median fold-change across the four groups. For comparisons between groups which displayed different population distributions based on positive and negative skewness, data were transformed by y = y^3^ prior to analysis (i.e., Fig. 3j only). AUC and fold-change graphs display the median plus 95% confidence interval. All relevant *p*-values, 95% CI and test performed are reported in the Results section. Only comparisons which were found to be statistically significant are indicated on the graphs, however all outlined comparisons in a multiple comparison were performed. The absence of significant *p*-values does not necessarily indicate that a biological effect or association does not exist, simply that there was not sufficiently strong quantitative evidence to statistically reject the null hypothesis. No adjustments for confounding factors/effect modifiers were made due to the exploratory nature of the outcomes, and the randomly selected small sample size (*n = 10/group*) aimed at a preliminary evaluation of cellular immune responses in humans.

### Role of funders

This research was funded in part by the Bill and Melinda Gates Foundation (BMGF). The funders did not play any role in study design, data collection, data analyses, interpretation, or writing of the manuscript.

## Results

The data presented in this manuscript represent tertiary exploratory T cell analyses performed on cryopreserved PBMC samples from a subset of volunteers (*n = 10* per group) enrolled in a completed Phase I clinical trial (ClinicalTrials.gov NCT03300050). A summary of the vaccine groups, intervals and analysis timepoints evaluated in this current manuscript is outlined in Table 1, and information on the demographics of participants selected for T cell assays is listed in Table 2. Safety data for these universal influenza virus vaccine candidates has previously been reported, where the vaccines were found to be safe and well-tolerated in humans.^13^^,^^14^

### Immunisation with cHA-based vaccines boosts HA stalk-specific T cells

In this study, we used the IFN-γ ELISpot assay to measure H1 stalk-specific T cells in cryopreserved PBMCs from individuals immunised with cHA-based universal influenza virus vaccines, or PLACEBO (Table 1). Data presented in Fig. 1a–e represent background subtracted IFN-γ ELISpot responses to pools of overlapping peptides corresponding to the H1 stalk domain of A/Michigan/45/2015 (see Supplementary Table S1). The HA stalk of A/Michigan/45/2015 has 98% amino acid sequence identity when compared with the H1 stalk of A/California/04/2009, present in the cHA vaccines used in this study. A timepoint of 7 days post-prime (D8), and 7 days post-boost (D92) was selected for T cell analysis, based on previous studies.^17^

In G1 participants (LAIV8-IIV5/AS03), we did not detect increases in HA stalk specific T cells post-prime (D8), as measured by spot-forming units (SFU) per 10^6^ PMBCs (Fig. 1a). However, a ∼3.7-fold-greater median HA stalk-specific T cell response was detected seven days following the boost immunisation with IIV5/ASO3, when compared with pre-boost (D92 versus D85 95% CI of the median: 218.30–970.00 versus 66.67–321.70 SFU/10^6^, Friedman test: *p = 0.019*). This corresponded to a ∼5.1-fold greater response compared to baseline (D92 versus D1 95% CI of the median: 218.30–970.00 SFU/10^6^ versus 58.33–245.00, Friedman test: *p = 0.0022*). Similar to G1 (LAIV8-IIV5/AS03), we did not detect increases in stalk-specific T cells in G2 (LAIV8-IIV5) participants following the LAIV8 prime (Fig. 1b). However, a small increase in the response was detected in this group following the IIV5 boost without adjuvant, with median HA stalk-specific T cells boosted ∼2.6-fold on D92 relative to D85 (95% CI of the median: 50.00–278.30 versus 9.17–185.00 SFU/10^6^, Friedman test: *p = 0.0016*). Participants in G4 received the IIV8/AS03-IIV5/AS03 vaccination regimen. These subjects had a ∼3.6-fold greater response in HA stalk-specific T cells post-prime (D8 versus D1 95% CI of the median: 145.00–690.00 versus 58.33–235.00 SFU/10^6^, Friedman test: *p = 0.0055*) when compared with baseline (Fig. 1c). A ∼1.8 fold-change was observed post-boost (D92) when compared with D85, although this was not statistically significant (95% CI of the median: 83.33–653.30 versus 35.00–283.30 SFU/10^6^, Friedman test: *p = 0.17*). However, when comparing D92 with baseline, a ∼3.3-fold greater median HA stalk-specific T cell response was observed (D92 versus D1 95% CI of the median: 83.33–653.30 versus 58.33–235.00 SFU/10^6^, Friedman test: *p = 0.036*). Subjects assigned to receive the PLACEBO (G3 and G5) did not show statistically significant changes in HA stalk-specific T cell responses following prime or boost vaccinations (Fig. 1d). The median response (SFU/10^6^ PBMCs) as presented in Fig. 1a–d is summarised for each group in Fig. 1e.

Area under the curve (AUC) analysis has been previously applied in immunological studies to assess the magnitude of an immune response within a defined time period.^17^^,^^69^ Therefore, we also compared the overall AUC for the HA stalk specific IFN-γ^+^ T cell response for each vaccine group from D1–D92 (Fig. 1f). When comparing between all vaccination groups, the median of the overall AUC (i.e., D1–D92) was ∼3.0-fold higher in the G4 (IIV8/AS03-IIV5/AS03) vaccinated group when compared with G2 (LAIV8-IIV5) (95% CI of median area: 9310–41,452 versus 2421–20,312, Kruskal–Wallis test: *p = 0.033*).

A ≥2-fold increase in antigen-specific T cell responses to influenza virus has previously been reported as a parameter for vaccine responsiveness.^53^^,^^70^^,^^71^ Therefore, we calculated the proportion of subjects with a ≥2-fold increase in T cell ELISpot response, indicated by a horizontal dashed grey line (Fig. 1g–i). G4 (IIV8/AS03-IIV5/AS03) performed best following the prime immunisation with IIV8/AS03 (Fig. 1g), with 7/10 individuals showing a ≥2-fold greater response (G4 versus G1: 95% CI of median fold change: 1.31–5.31 versus 0.52–1.94 fold-change, Kruskal–Wallis: *p = 0.034* and G4 versus G2: 95% CI of median fold change: 1.31–5.31 versus 0.25–2.20 fold-change, Kruskal–Wallis: *p = 0.018*). There was minimal vaccine responsiveness post-prime in the other vaccination groups. G1 (LAIV8-IIV5/AS03) showed the greatest responsiveness in the stalk-specific response upon boosting with IIV5/AS03 (Fig. 1h), with a ≥2-fold response in HA stalk-specific T cell responses in 8/9 individuals at D92 versus D85 (95% CI of median fold change: 2.08–4.69 versus 0.46–2.67 fold-change when compared with PLACEBO, Kruskal–Wallis: *p = 0.0081*). In G2 (LAIV8-IIV5), 5/10 subjects had ≥2-fold responses, and in G4, 4/10 had ≥2-fold responses following receipt of the IIV5/AS03 boost (D92 versus D85). However, it is worth noting that the median ELISpot response at D85 for G4 was higher than other groups post-prime, which may have affected subsequent fold-changes following boost (Fig. 1e). When comparing the final timepoint to baseline (D92 versus D1), 8/10 individuals in G1 (LAIV8-IIV5/AS03) had a ≥2-fold stalk-specific T cell response (Fig. 1i). A total of 5/10 volunteers had a ≥2-fold stalk-specific T cell responses for G2 (LAIV8-IIV5) and for G4 (IIV8/AS03-IIV5/AS03). Subjects assigned to receive the PLACEBO group did not show statistically significant increases in HA stalk-specific T cell responses following prime or boost vaccinations (Fig. 1d), although some PLACEBO participants had ≥2-fold responses post-prime (D8; 3/10 volunteers), post-boost (D92; 2/8 volunteers), or when comparing D92 to D1 (2/8 volunteers).

A factor which may contribute to bias in the response to immunisation could include prior exposure to influenza virus and the magnitude of pre-existing T cell responses at baseline. At baseline (pre-immunisation), H1 stalk-specific T cells were present at detectable levels in most volunteers, with median responses of ∼86 SFU/10^6^ PBMCs (Fig. 1j). Importantly however, no statistically significant differences were observed in baseline HA stalk-specific T cell responses between subjects between groups (Kruskal–Wallis test: *p = 0.46*).

### Immunisation with cHA-based vaccines expands the breadth of cellular reactivity against the conserved HA stalk domain

**Fig. 2:**
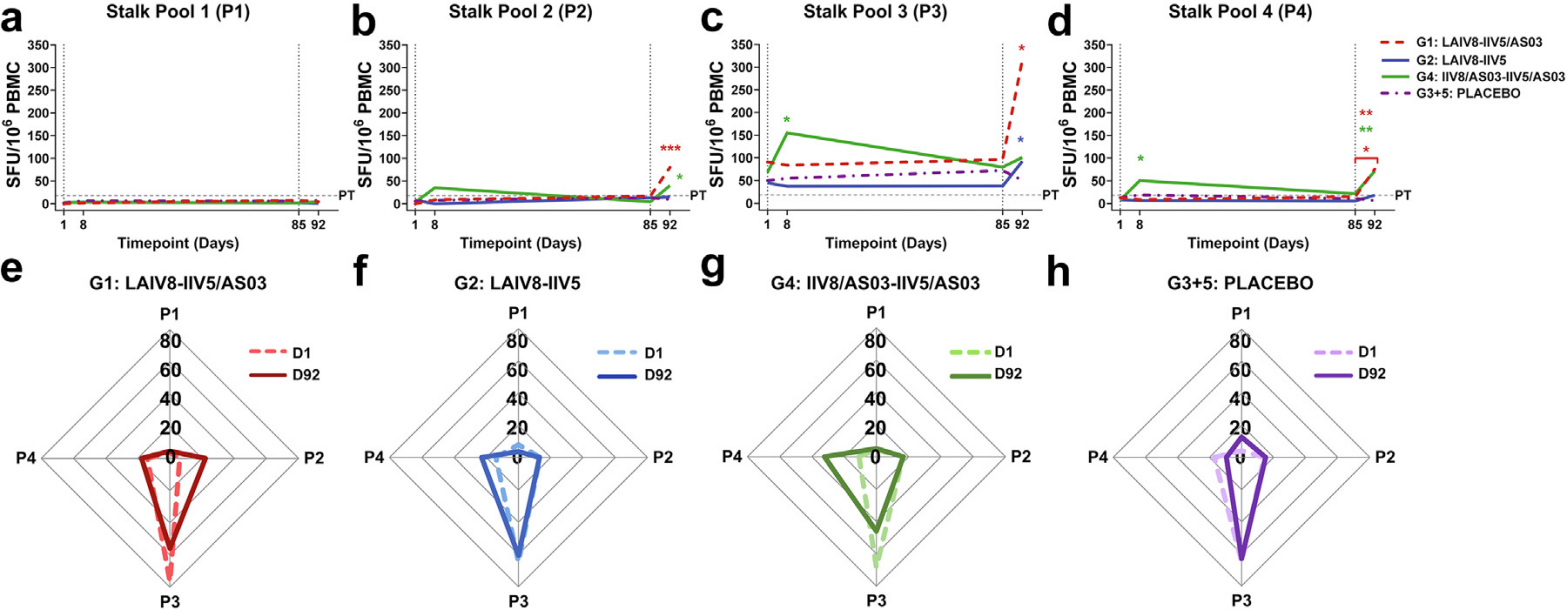
**Breadth of the HA stalk-specific T cell response against defined peptide pools following immunisation with cHA-based universal influenza virus vaccine candidates.** Cryopreserved human PBMCs obtained prior to immunisation (D1), seven days post-prime (D8), pre-boost (D85) and seven days post-boost (D92) were stimulated with pools of peptides corresponding to HA stalk from A/Michigan/45/2015, and IFN-γ secretion detected by ELISpot (*n = 10/group*, single biological replicate from duplicate/triplicate wells). **(a–d)** Median HA stalk-specific T cell responses to each peptide pool (P1–P4) expressed as SFU/10^6^ PBMCs for each volunteer. Dashed vertical grey line indicates prime (D1) and boost (D85) immunisation timepoints, and horizontal dashed grey line indicates the positive threshold (PT), which represents the median+2X median absolute deviation (MAD) for DMSO baseline control wells (18 SFU/10^6^ PBMCs). Statistical analysis on intra-group paired data (i.e., different timepoints for each individual volunteer) was performed using the non-parametric Friedman test with Dunn’s correction for multiple comparisons. Volunteers which had missing timepoints were excluded from the Friedman analysis, but due to the limited sample size, all volunteer responses are represented graphically. Exclusions due to isolated missing timepoints are outlined in Table 1. Statistical significance icons are shown on the graph as ∗*p < 0.05*, ∗∗*p < 0.01*, ∗∗∗*p < 0.001*. Data shown in for P2 **(b)** denotes a statistically significant difference between D92 versus D1 (∗∗∗*p = 0.0004*) for G1 (LAIV8-IIV5/AS03), or D92 versus D85 (∗*p = 0.036*) for G4 (IIV8/AS03-IIV5/AS03). **(c)** In P3, a statistically significant difference between D92 versus D1 (∗*p = 0.024*) for G1 (LAIV8-IIV5/AS03), or between D92 and D85 (∗*p = 0.028*) for G2 (LAIV8-IIV5) are shown. Responses for G4 (IIV8/AS03-IIV5/AS03) show a statistically significant difference between D8 and D1 (∗*p = 0.028*). **(d)** In P4, a statistically significant difference between D92 and D85 (∗*p = 0.032*), and D92 versus D1 (∗∗*p = 0.0016*) for G1 (LAIV8-IIV5/AS03) are shown. Responses for G4 (IIV8/AS03-IIV5/AS03) also show a statistically significant difference between D8 versus D1 (∗*p = 0.046*) and D92 versus D1 (∗∗*p = 0.0073*). **(e–h)** Radar charts show the proportion of the response to each HA stalk pool (P1–P4) expressed as a percentage (%) of the total summed HA-stalk response, where the total response in each pool is 100%. These figures display changes in the relative response to distinct peptide pools, not the overall magnitude of the response.

A gap in our knowledge has been in the identification of specific T cell epitopes in the HA stalk, and in understanding their precise functional roles in influenza virus infection and/or vaccination. Selected studies have undertaken epitope mapping studies to identify T cell “hot zones” or “dead zones” in the HA stalk.^72^ By breaking down the HA stalk into distinct peptide pools for stimulation, we were able to identify specific sub-regions of the HA stalk for which volunteers had higher T cell reactivity at baseline, and following immunisation with cHA-based IIV/AS03 vaccines (Fig. 2a–d).

**Fig. 3:**
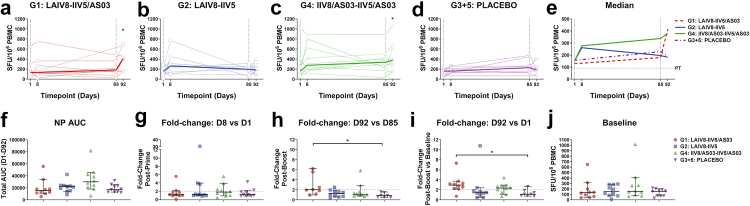
**NP-specific T cell responses following immunisation with cHA-based universal influenza virus vaccine candidates.** Cryopreserved human PBMCs obtained prior to immunisation (D1), seven days post-prime (D8), pre-boost (D85) and seven days post-boost (D92) were stimulated with pools of overlapping peptides corresponding to NP from A/Michigan/45/2015, and IFN-γ secretion measured by ELISpot. **(a–d)** Time course of individual NP-specific T cell responses presented as SFU/10^6^ PBMCs for each volunteer (*n = 10/group*, single biological replicate from duplicate/triplicate wells). The median is shown as a heavy line. Statistical analyses on intra-group paired data (i.e., different timepoints for each individual volunteer) were performed using the non-parametric Friedman test with Dunn’s correction for multiple comparisons. Volunteers with missing timepoints were excluded from the Friedman analysis, but due to the limited sample size, all volunteer responses are represented graphically. Exclusions due to isolated missing timepoints are outlined in Table 1. Statistical significance icons are shown on the graph as ∗*p < 0.05*, ∗∗*p < 0.01*, ∗∗∗*p < 0.001*. Icons denote statistically significant differences between **(a)** D92 and D1 (∗*p = 0.047*) for G1 (LAIV8-IIV5/AS03), and **(c)** D92 against D1 (∗*p = 0.028*) for G4 (IIV8/AS03-IIV5/AS03). **(e)** Median NP-specific IFN-γ ELISpot response for each group. Dashed vertical grey line indicates prime (D1) and boost (D85) immunisation timepoints, and horizontal dashed grey line indicates the positive threshold (PT) for summed NP responses, which represents the median+2X median absolute deviation (MAD) for DMSO baseline control wells corrected for summed pools (PT = 92 SFU/10^6^ PBMCs). **(f)** Total area under the curve (AUC) for NP-specific IFN-γ ELISpot response from D1–D92. **(g)** Fold-change in individual IFN-γ responses post-prime, D8 versus D1, for each vaccine group. **(h)** Fold-change in individual IFN-γ responses post-boost (D92 versus D85) for each vaccine group. Inter-group comparisons were analysed using the Kruskal–Wallis test with Dunn’s correction for multiple comparisons (∗*p = 0.017*). **(i)** Fold-change in individual IFN-γ responses at D92 versus D1 for each vaccine group. Inter-group comparisons were analysed using the Kruskal–Wallis test with Dunn’s correction for multiple comparisons *∗ p = 0.027*. **(j)** Baseline responses to NP peptides in each vaccine group. Solid line denotes median with 95% confidence intervals (CI) for **f–j**. Dashed grey line indicates ≥2-fold elevated T cell responses after vaccination for **g–i**.

We detected little response at baseline to pool 1 (P1), pool 2 (P2) or pool 4 (P4), and determined that baseline IFN-γ^+^ ELISpot responses were predominantly (60–80%) skewed towards peptides in P3 (Fig. 2c). Prime immunisation with LAIV8 in both G1 and G2 did not boost HA stalk responses to any pool. However, for G4 (IIV8/AS03-IIV5/AS03), upon prime immunisation with IIV8/AS03 (Fig. 2c and d), the response to peptide pools P3 and P4 was ∼2.3-fold or ∼6.8-fold greater on D8 versus D1 respectively (**P3:** 95% CI of median: 73.33–316.70 versus 30.00–115.00 SFU/10^6^, Friedman test: *p = 0.028,* and **P4:** 95% CI: 5.00–246.70 versus 3.33–18.33 SFU/10^6^, Friedman test, *p = 0.046*). A boost with IIV5 alone did little to increase responses to the HA stalk (i.e., G2), although responses to P3 were ∼2.4-fold greater on D92 as compared with D85 (95% CI of the median: 35.00–213.3 versus 0.83–158.3 SFU/10^6^, Friedman test: *p = 0.028*). In contrast, in G1 (LAIV8-IIV5/AS03), responses were boosted against P2, P3 and P4 epitopes upon subsequent immunisation with the IIV5/AS03 vaccine when comparing D92 to D85 (Fig. 2b–d). In P2, at D92 versus D1 a predominantly *de novo* response was elicited (95% CI of the median: 25.00–190.00 versus 0.00–15.00 SFU/10^6^, Friedman test, *p = 0.0004*)*.* In P3, responses were ∼4.0-fold greater at D92 versus D1 (95% CI of the median: 105.80–541.70 versus 28.33–183.30 SFU/10^6^, Friedman test, *p = 0.024*). In P4, responses were ∼5.2-fold greater at both D92 versus D85 (95% CI of the median: 32.50–241.70 versus 10.00–51.67 SFU/10^6^, Friedman test, *p = 0.032*), and D92 versus D1 (95% CI: 32.50–241.70 versus 5.00–31.67 SFU/10^6^, Friedman test, *p = 0.0016*). In G4 (IIV8/AS03-IIV5/AS03), the boost with IIV5/AS03 resulted in a ∼9.6-fold greater response to peptides in P2 when comparing D92 to D85 (Fig. 2b: 95% CI of median: 1.67–76.67 versus 0.0–40.0 SFU/10^6^, Friedman test: *p = 0.036*), and a ∼9.6-fold greater response to peptides in P4, when comparing D92 to D1 (Fig. 2d; 95% CI of median: 30.00–203.30 versus 3.33–18.33 SFU/10^6^, Friedman test: *p = 0.0073*). The P3 peptides span a region of the HA stalk which includes the long alpha helix (LAH), a highly conserved region within the HA stalk domain (Supplementary Table S1), and the P4 pool contains peptides within the transmembrane domain and cytoplasmic tail of HA. Previous studies measuring T cell responses to HA in humans have also reported responses which map to the corresponding P3 and P4 pool described in this study.^54^^,^^73^^,^^74^

We plotted radar charts to summarise changes in the proportion of the response to each HA stalk pool (P1-4), which are shown as a percentage of the total summed response (Fig. 2e–h). When comparing the final post-boost timepoint to baseline (D92 versus D1), we observed that G1 (LAIV8-IIV5/AS03) participants displayed broadening in the overall response as follows: P2 responses were increased from 6% to 22%, and baseline dominance towards P3 was reduced from 76% to 55% (Fig. 2e). Similarly, in G4 participants (IIV8/AS03-IIV5/AS03), we also observed altered breadth: baseline dominance towards P3 responses decreased from 68% to 45%, and P4 responses increased from 11% to 32%.

### Immunisation with cHA-based vaccines boosts T cell responses to internal nucleoprotein

Influenza virus-specific, and particularly NP-specific T cells, have previously been proposed as a correlate of protection against disease severity and/or virus shedding and transmission.^55^^,^^57^^,^^58^^,^^75^^,^^76^ Therefore, we measured IFN-γ ELISpot responses against a pool of peptides corresponding to NP following immunisation with cHA IIV and LAIV-based universal influenza virus vaccines (Fig. 3a–i). There were minimal changes in NP-specific T cell responses following the prime immunisation with LAIV8 on D8, for G1 and G2 (Fig. 3a and b). Similarly, G4 (IIV8/AS03-IIV5/AS03) immunised individuals did not show significant boosting of NP-specific T cells post-prime (Fig. 3c). No significant boosting of NP-specific T cells was observed in PLACEBO recipients at any timepoint (Fig. 3d). Median responses (SFU/10^6^ PBMCs) as presented in Fig. 3a–d are summarised for each group in Fig. 3e. Following the boost immunisation with IIV5/AS03, G1 participants showed a ∼2.9-fold greater NP-specific T cell response when comparing D92 to baseline D1 (Fig. 3a and 95% CI of median: 149.20–715.00 versus 40.00–646.70 SFU/10^6^, Friedman test: *p = 0.047*). Responses were not increased for G4 (IIV8/AS03-IIV5/AS03), when comparing D92 to D85 (Fig. 3c). However, when comparing D92 to baseline, responses were ∼2.4 fold greater (D92 versus D1 95% CI of median: 185.00–695.00 versus 75.00–413.30 SFU/10^6^, Friedman test: *p = 0.028*).

Once again, we compared the overall AUC (D1–D92) for the NP-specific IFN-γ^+^ T cell response for each vaccine group (Fig. 3f). When comparing between all vaccination groups, the overall AUC response to NP was highest for G4 (IIV8/AS03-IIV5/AS03). We also applied a threshold of ≥2-fold increase in antigen-specific T cell responses as a parameter for responsiveness to NP (Fig. 3g–i). Post-prime, only 2/10 participants in G1 (LAIV8-IIV5/AS03) had a ≥2-fold change in their NP-specific T cell response, whereas this increased to 4/8 individuals post-boost, and to 9/10 individuals when comparing D92 to D1 (Fig. 3g–i). Post-boost (D92 versus D85), responses were increased over PLACEBO (Fig. 3h: 95% CI of median fold change: 0.98–6.23 versus 0.47–1.59, Kruskal–Wallis: *p = 0.017*. Fig. 3i represents the response between D92 and D1 (95% CI of median fold change: 2.16–3.73 versus 0.74–2.66, Kruskal–Wallis: *p = 0.027*)). For G2 (LAIV8-IIV5), only 3/10 individuals showed a ≥2-fold increase on D8 versus D1, only 1/9 when comparing D92 to D85, and only 2/10 when comparing D92 to D1 (Fig. 3g–i). For G4 participants (IIV8/AS03-IIV5/AS03), only 4/10 subjects had ≥2-fold greater NP-specific T cell responses (Fig. 3g). Boosting with IIV5/AS03 did not increase NP-specific T cell responses (D92 versus D85), with only 2/10 individuals having responses ≥2-fold when comparing D92 to D85 (Fig. 3h). However, when comparing the D92 timepoint with baseline (Fig. 3i), 7/10 individuals in G4 (IIV8/AS03-IIV5/AS03) had NP-specific T cell responses which had been boosted ≥2-fold. Although no significant boosting of NP-specific T cells was observed in participants who received the placebo (Fig. 3d), selected individuals did have responses which were increased ≥2-fold (2/10 at D8 and 1/8 at D92 versus baseline).

A factor which may contribute to bias in the response to immunisation could include prior-exposure to influenza virus and the magnitude of pre-existing T cell responses at baseline. To consider this, we determined that prior to vaccination (i.e., baseline), NP-specific T cells were present at detectable levels in all volunteers, with median responses of ∼145 SFU/10^6^ PBMCs (Fig. 3j). Importantly, no differences were observed in baseline NP-specific T cell responses between groups (Kruskal–Wallis test of transformed data: *p = 0.69*).

### Breadth of NP-specific T cell responses following immunisation with cHA-based universal influenza virus vaccines

**Fig. 4:**
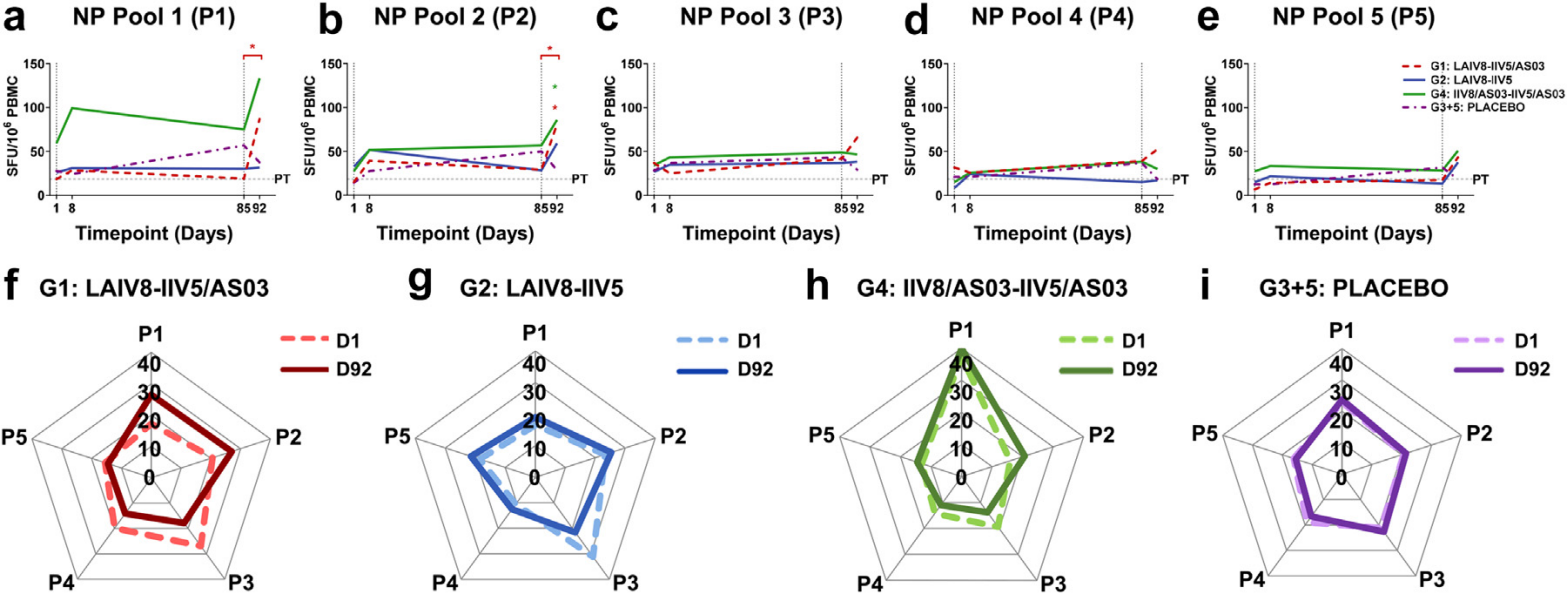
**Breadth of the NP-specific T cell response against defined peptide pools following immunisation with cHA-based universal influenza virus vaccine candidates.** Cryopreserved human PBMCs obtained prior to immunisation (D1), seven days post-prime (D8), pre-boost (D85) and seven days post-boost (D92) were stimulated with pools of peptides corresponding to NP from A/Michigan/45/2015, and IFN-γ secretion detected by ELISpot (*n = 10/group*, single biological replicate from duplicate/triplicate wells). **(a–e)** Median NP-specific T cell responses to each peptide pool (P1–P5) expressed as SFU/10^6^ PBMCs for each volunteer. The dashed vertical grey line indicates prime (D1) and boost (D85) immunisation timepoints, and horizontal dashed grey line indicates the positive threshold (PT), which represents the median+2X median absolute deviation (MAD) for DMSO baseline control wells (18 SFU/10^6^ PBMCs). Statistical analysis on intra-group paired data (i.e., different timepoints for each individual volunteer) was performed using the non-parametric Friedman test with Dunn’s correction for multiple comparisons. Volunteers which had missing timepoints were excluded from the Friedman analysis, but due to the limited sample size, all volunteer responses are represented graphically. Exclusions due to isolated missing timepoints are outlined in Table 1. Statistical significance icons are shown on the graph as ∗*p < 0.05*. **(a)** Data shown for P1 denotes a statistically significant difference between D92 and D85 (∗*p = 0.027*) for G1 (LAIV8-IIV5/AS03). **(b)** Responses to P2 show a statistically significant difference between D92 and D85 (∗*p = 0.036*) and D92 versus D1 (∗*p = 0.036*) for G1 (LAIV8-IIV5/AS03), or D92 and D1 (∗*p = 0.028*) for G4 (IIV8/AS03-IIV5/AS03). **(f–i)** Radar charts show the proportion of the response to each NP pool (P1-5) expressed as a percentage (%) of the total summed NP response, where the total response in each pool is 100%. These figures display changes in the relative response to distinct peptide pools, not the overall magnitude of the response.

When NP-specific responses were broken down by NP peptide pool (P1–P5), limited responses were observed following the prime immunisation (Fig. 4a–e). Although we did observe an increase in the NP response to the P1 pool in G4 (IIV8/AS03-IIV5/AS03) participants following prime immunisation with IIV8/AS03 (Fig. 4a), this was not statistically significant. We also observed ∼1.5-fold greater responses to P2 peptides in G4 (IIV8/AS03-IIV5/AS03) when comparing D92 with D85 following IIV5/AS03 boosting (Fig. 4b), but again, this was not significant. In contrast, following boost vaccination of G4 with IIV5/AS03 (IIV8/AS03-IIV5/AS03) on D92 as compared with D1, we observed ∼3.1-fold greater responses in P2 (95% CI of median: 16.67–103.30 versus 6.67–48.33 SFU/10^6^, Friedman test: *p = 0.028*). In G1 (LAIV8-IIV5/AS03) participants, increases in the response against peptides mapping to NP pools P1 and P2, were detected (Fig. 4a and b). Responses to P1 peptides were ∼5.5-fold greater (95% CI: 13.33–413.30 versus 5.00–140.00 SFU/10^6^, Friedman test: *p = 0.027*) when comparing D92 with D85. P2 responses were ∼2.7-fold greater (95% CI: 14.17–200.00 versus 0.00–101.70 SFU/10^6^, Friedman test: *p = 0.036*) or ∼3.6-fold greater (95% CI: 14.17–200.00 versus 0.00–143.30 SFU/10^6^, Friedman test, *p = 0.036*) greater, when comparing D92 with D85, or D92 with D1 respectively.

Again, we plotted radar charts to represent changes in the proportion of the response to each of the five NP peptide pools (P1-5) for each group (Fig. 4f–i). For G1 (LAIV8-IIV5/AS03), there were some changes in the proportion of the response when comparing D92 to baseline: with P1 responses increased from 17% to 24%, P2 responses increased from 21% to 27%, responses to P3 epitopes decreased from 27% to 18% and P4 responses decreased 20%–14%. Responses to P5 peptides remained unchanged. In G2 (LAIV8-IIV5), there were minor increases in responses in P1, P2, P4 and P5 whereas P3 responses decreased from 31% to 21%. In G4 (IIV8/AS03-IIV5/AS03), we also observed some changes when comparing D92 to baseline: with P1 responses increased from 37% to 40%, P2 responses increased from ∼16% to 20%, responses to P3 epitopes decreased from ∼19% to 14%, and P4 responses decreased from ∼14% to 11%.

## Discussion

The ongoing threat of an influenza virus pandemic, as exemplified by global spread of zoonotic H5N1 infections, and human infections with H5N1 (e.g., Cambodia, South America and USA),^77^, ^78^, ^79^, ^80^, ^81^ highlights the need for vaccines which elicit broad protection across different strains and subtypes. Vaccines targeting conserved influenza virus antigens, which are capable of eliciting both humoral and cellular immune responses simultaneously, would be desirable as a universal influenza virus vaccine. In this study, we expand analysis of an adjuvanted cHA immunisation regimen shown to boost cross-reactive HA stalk Abs in humans,^13^^,^^14^ to evaluate T cell responses. Our analysis reveals that immunisation with adjuvanted cHA-based IIV can also successfully boost HA stalk-specific T cell responses in humans.

T cells have long been identified as key modulators of disease. In both human influenza virus challenge studies, and prospective cohort studies of natural community-acquired infection, T cells have been associated with reduced viral shedding and the reduction of symptom severity.^55^^,^^57^^,^^58^^,^^70^ Across these studies, different CD4^+^ and CD8^+^ T cell subsets targeting NP, M1 or PB1 have been identified as correlating with protection.^55^^,^^57^^,^^58^^,^^75^ T cells can have diverse protective functions, from cytolytic activity, to aiding the recruitment of innate effector cells to the lung, as well as providing T cell help in the generation of Abs.^12^^,^^82^^,^^83^ Indeed, T helper cell responses and Ab responses have previously been reported to correlate in clinical studies of influenza virus vaccination or infection.^84^^,^^85^

Limited CD8^+^ T cell responses against HA, and particularly the HA stalk, have been reported in mice following influenza virus infection and vaccination.^35^^,^^86^, ^87^, ^88^, ^89^ The paucity of published data on T cell epitopes in the HA stalk, specifically CD8^+^ T cell epitopes, highlights an area of research need in the field of HA-stalk based universal vaccines. Some prior studies have demonstrated that T cell epitopes in the immunosubdominant HA stalk can be targeted by vaccination in humans. A *Phase I* and *II* evaluation of a universal influenza virus vaccine candidate, Multimeric-001, identified an MHC class II epitope in the stalk of H3.^90^, ^91^, ^92^ This epitope was also independently identified as a CD4^+^ T cell epitope following natural influenza virus infection.^93^ Furthermore, a H5-stalk based CD4^+^ T cell epitope was also identified from a natural infection study.^54^ Interestingly, the latter study failed to detect any human CD8^+^ T cell epitopes in the H5 head or stalk.^54^ In support of this, another study describing UK and Vietnamese cohorts exhibiting memory T cell responses to NP were found to map to both CD4^+^ and CD8^+^ T cells, whereas HA- and NA-specific memory T cell responses identified were solely restricted to CD4^+^ T cells.^54^

The immunodominance of the HA head domain ensures that sequential immunisation with conventional seasonal IIVs would result in largely strain-specific immune responses directed towards the HA head. In contrast, the selection of cHA-based vaccines for sequential immunisation is an approach which has been shown to refocus immunity towards the HA stalk. However, an alternative to the use of cHA-based IIVs for HA stalk re-focusing could include immunisation regimens with antigenically distinct pandemic IIVs, such as a prototype H5N1 IIV. A prior study evaluated a two-dose regimen with an AS03-adjuvanted H5N1 split virion vaccine, and determined that H5-specific CD4^+^ T cell responses were present at baseline, and these could be boosted upon immunisation (NCT00309634).^62^ Although the authors did not formally measure HA stalk-specific cellular immunity, and used a different assay (flow cytometry), it is possible that cross-reactive stalk T cells were also boosted using this immunisation regimen.

A strength of this report is that it describes and maps HA stalk-specific T cell responses down to the sub-domain/pool level in humans following immunisation with a universal influenza vaccine candidate. However, although we detected boosting of HA-stalk reactive T cells in this study, a caveat of our findings is that we specifically measured responses by *ex vivo* IFN-γ ELISpot assay, and cannot distinguish between CD4^+^ and CD8^+^ T cells. Nonetheless, a discussion of responses to defined HA stalk peptide pools used in our study with those reported in the literature could be informative to the field. For HA-specific CD4^+^ T cell responses measured against H5 by Lee and colleagues in the natural infection UK/Vietnamese study, T cell epitopes were identified in three regions of the HA stalk.^54^ Of those HA stalk epitopes, one epitope maps to HA stalk pool 2 (P2) in our study. Several other studies also identified CD4^+^ T cell epitopes mapping to this “P2” HA stalk region.^73^^,^^74^^,^^94^^,^^95^ The other two epitopes from the UK/Vietnamese study map to HA stalk pool 3 (P3) in our study. Interestingly, at baseline, T cell responses directed towards epitopes in HA stalk P3 represented ∼60–80% of the response across all groups, and this pool was also where the majority of the T cell expansion was measured following the boost immunisation of G1 (LAIV8-IIV5/AS03) and G4 (IIV8/AS03-IIV5/AS03) with IIV5/AS03. P3 contains the long alpha helix (LAH), where CD4^+^ T cell responses have been mapped to in both mice^95^^,^^96^ and humans after natural influenza virus infection or vaccination.^73^^,^^74^ It is suggested that this region is a hotspot for T cell epitopes due to the stability of the tertiary structure and the accessibility to proteasomal processes required for generation of peptide epitopes.^72^ In addition to responses targeting P3, immunisation of G1 and G4 with IIV/AS03 led to broadening of the T cell response against HA stalk peptide pool 4 (P4). This pool corresponds to the membrane proximal, carboxyl terminal region of the HA stalk domain that contains the transmembrane domain (TM) and cytoplasmic tail. Using a tetramer-guided epitope mapping approach, two human CD4^+^ T cell epitopes with HLA-DR restriction have been identified in the final membrane-proximal residues of the HA stalk, extending into the transmembrane domain, and a further CD4^+^ epitope-rich area in amino acid sequences spanning HA stalk P3 and P4 in our study.^74^ Given the high sequence similarity of the TM domain within H1 and H3 viruses respectively, inducing responses against this region may be beneficial for broad cellular reactivity.^97^

An epitope mapping study of donor PBMCs previously identified CD4^+^ T cell responses mapping across the full NP antigen, with relatively equal responses across all epitopes identified, including at NP 19–42 which would correspond to NP P1, and NP 97–120 which corresponds to a region spanning NP P1 and P2.^50^ CD8^+^ T cell epitopes within NP have also been experimentally confirmed throughout the antigen,^98^ with one study mapping CD8^+^ NP-specific responses in 5 PBMC donors across NP with 6 hotspots identified.^99^ A similar study conducted using non-HLA-A2 PBMC donors revealed comparable results.^51^ Across these two prior studies, the majority of the immunodominant epitopes were clustered at the carboxyl terminal 2/3 of the NP protein (NP 140–412), which does not include peptides in our NP P1 pool. Therefore, we speculate that the responses we measured against NP P1 are more likely to be CD4^+^ T cell epitopes, although we have not verified this experimentally. Unlike subunit vaccines, split virion vaccines, such as conventional IIV and the universal cHA-based vaccines described in this study may contain residual NP, although this is likely manufacturer and batch-dependent.^100^^,^^101^ As such, some boosting of immunity to NP can be observed, albeit at a lower level than observed following natural infection.^102^

We consider and acknowledge both the limitations and strengths of our exploratory study. The methodological constraints encompass a limited sample size per group, potential for uncontrolled or unmeasured confounding factors in between-group comparisons, and susceptibility to regression towards the mean within groups. In addition, despite this being an exploratory study, other factors related to confounding or bias, such as sex, age or ethnicity may have affected our findings as a result of the small sample size. To acknowledge this, we have outlined the demographic characteristics of the participants in our randomly sampled subset (Table 2). Although statistically significant changes in immune responses at various timepoints were observed, substantial confidence intervals (95%) are evident for many comparisons which may impact the clinical interpretation and importance, a limitation of performing this study with a small sample size. The main laboratory limitation lies in the T cell responses not being distinguished into their respective CD4^+^ or CD8^+^ subpopulations. Downstream mucosal homing markers, effector identities and memory phenotypes were also not characterised, which would permit more extensive interpretation of the data. PBMC sampling only represents cells present in the peripheral blood, so germinal centre T follicular helper cells and T cells resident in the respiratory mucosa were not sampled. Furthermore, we have measured just a single cytokine, IFN-γ, so cannot comment on the polyfunctionality of the T cell responses described. In addition, due to the small sample size per group, as a result of limited sample availability, we do not possess sufficient power to correlate HA stalk-specific T cell responses with previously reported stalk-specific Ab responses on a per volunteer basis. An extrapolation of the role of cellular immunogenicity to protection is challenging, as the field lacks formal correlates of protection for influenza virus for this arm of the immune response. Additionally, the types of assays employed, and parameters measured for T cell assays in clinical studies are not standardised, making direct comparisons with other studies difficult.^8^^,^^103^

A major strength of our study is that it is to date the most substantial HA stalk-targeting immunisation cohort combining multiple routes of administration, ± adjuvant, with T cell responses described at multiple timepoints pre-/post-vaccination. As more studies of HA-stalk targeting universal influenza virus vaccine candidates progress clinically with larger sample sizes per group, the level of detail on specific epitopes, phenotypes and functions associated with the induced T cell response will grow, building upon the foundation data presented in this study. Later phase clinical studies may elucidate the protective role of these T cell populations, where currently there is a paucity of data. Preliminary studies such as this, will enable parallels to be drawn between clinical and pre-clinical data and support the ongoing, more detailed cellular immunophenotyping in future clinical vaccine trials. In summary, in this study we have demonstrated that adjuvanted cHA-based IIVs are capable of stimulating/boosting HA stalk-specific T cell responses in humans. It has previously been shown that these adjuvanted cHA-based IIV candidates induce durable, HA stalk-specific Abs in humans which are broad in terms of their breath of reactivity against group 1 HAs from H1 clade (H1, H2, H5 and H6), the H9 clade (H8, H9 and H12) and the bat HAs (H17 and H18),^44^ and elicit diverse functional activities (neutralisation, Fc effector function activation and *in vivo* protection).^13^ Further investigation of the role of defined T cell populations, or specific T cell epitopes in the HA stalk domain in protection following immunisation with universal influenza virus vaccine candidates, is warranted.

## Contributors

Conceptualization, LC, CMB and RN; methodology, LC, CMB and JTG; validation, LC and CMB; formal analysis, LC, and CMB; investigation, LC, CMB and JTG; resources, FK, PP and LC; writing—original draft, CMB and LC; writing—review and editing, all authors contributed to review and editing of the final draft, and all authors read and approved the final version of the manuscript; visualization, CMB and LC; supervision, LC and FK; funding acquisition, LC, FK, PP, AG-S. LC and CB directly accessed and verified the underlying data reported in the manuscript.

## Data sharing statement

The clinical study protocol is available online at https://clinicaltrials.gov/study/NCT03300050. All raw generated in this study have been submitted to the Immunology Database and Analysis Portal (ImmPort https://www.immport.org/shared/home), a NIAID Division of Allergy, Immunology and Transplantation funded data repository under Study Accession SDY2509 under the User Defined ID: Study-198_Chimeric-NP, and will be openly accessible from the date of publication at https://www.immport.org/shared/study/SDY2509.

## Declaration of interests

The Icahn School of Medicine at Mount Sinai (ISMMS) has filed patent applications regarding universal influenza virus vaccines naming AGC, PP, RN and FK as inventors. AGC, PP and FK have also received royalties and research support for their laboratories from GSK in the past and are currently receiving research support from Dynavax for development of influenza virus vaccines. FK has consulting agreements with Pfizer, GSK, Third Rock Ventures and Avimex.

The AG-S. laboratory has also received research support from Pfizer, Senhwa Biosciences, Kenall Manufacturing, Blade Therapeutics, Avimex, Johnson & Johnson, 7Hills Pharma, Pharmamar, ImmunityBio, Accurius, Nanocomposix, Hexamer, N-fold LLC, Model Medicines, Atea Pharma, Applied Biological Laboratories and Merck, outside of the reported work. A.G.-S. has consulting agreements for the following companies: Esperovax, Farmak, Applied Biological Laboratories, Pharmamar, 7Hills Pharma, Avimex, Paratus, Synairgen, Accurius, Pfizer and Nanocomposix outside of the reported work. A.G.-S has consulting agreements for the following companies involving cash and/or stock: Castlevax, Amovir, Vaxalto, Pagoda, Contrafect, CureLab Oncology, CureLab Veterinary and Vivaldi Biosciences outside of the reported work. A.G.-S. has been an invited speaker in meeting events organised by Seqirus, Janssen, Abbott and AstraZeneca. JTG is an employee of Argenx US. RN is an employee and shareholder of Moderna. DIB serves on the Data Safety Monitoring Board and Advisory Board for Moderna. AN is an employee of GSK and has vested stocks in GSK. EBW has received funding support from Pfizer, Moderna, Sequiris, Najit Technologies Inc, Leidos Biomedical and Clinetic for the conduct of clinical trials and clinical research. EBW has served as an advisor to Vaxcyte, a consultant to ILiAD biotechnologies and a data safety monitoring board member for Shionogi.
