# Supplementary material for: A chimeric haemagglutinin-based universal influenza virus vaccine boosts human cellular immune responses directed towards the conserved haemagglutinin stalk domain and the viral nucleoprotein
Source: eBioMedicine. 2024 May 27;104:105153. doi: 10.1016/j.ebiom.2024.105153 (PMC11154122; doi:10.1016/j.ebiom.2024.105153)
Supplement: Supplementary Tables S1–S3 [file mmc1.docx]

**TABLE S1. HA Stalk Peptide Pool (H1 Stalk A/Michigan/45/2015)**

| **Pool (P1-P4)** | **Peptide #** | **Length** | **Amino Acid Sequence** |
| --- | --- | --- | --- |
| **P1** | HA1-1.1 | 15 | MKAILVVLLYTFTTA |
| **P1** | HA1-1.2 | 15 | VVLLYTFTTANADTL |
| **P1** | HA1-2 | 20 | TFTTANADTLCIGYHANNST |
| **P1** | HA1-3 | 20 | CIGYHANNSTDTVDTVLEKN |
| **P1** | HA1-4 | 20 | DTVDTVLEKNVTVTHSVNLL |
| **P1** | HA1-5 | 19 | VTVTHSVNLLEDKHNGKLC |
| **P2** | HA2-1 | 20 | CNTTCQTPEGAINTSLPFQN |
| **P2** | HA2-2 | 20 | AINTSLPFQNIHPITIGKCP |
| **P2** | HA2-3 | 20 | IHPITIGKCPKYVKSTKLRL |
| **P2** | HA2-4 | 20 | KYVKSTKLRLATGLRNVPSI |
| **P2** | HA2-5 | 20 | ATGLRNVPSIQSRGLFGAIA |
| **P2** | HA2-6 | 20 | QSRGLFGAIAGFIEGGWTGM |
| **P2** | HA2-7 | 20 | GFIEGGWTGMVDGWYGYHHQ |
| **P2** | HA2-8 | 20 | VDGWYGYHHQNEQGSGYAAD |
| **P2** | HA2-9 | 20 | NEQGSGYAADLKSTQNAIDK |
| **P3** | HA2-10 | 20 | LKSTQNAIDKITNKVNSVIE |
| **P3** | HA2-11 | 20 | ITNKVNSVIEKMNTQFTAVG |
| **P3** | HA2-12 | 20 | KMNTQFTAVGKEFNHLEKRI |
| **P3** | HA2-13 | 20 | KEFNHLEKRIENLNKKVDDG |
| **P3** | HA2-14 | 20 | ENLNKKVDDGFLDIWTYNAE |
| **P3** | HA2-15 | 20 | FLDIWTYNAELLVLLENERT |
| **P3** | HA2-16 | 20 | LLVLLENERTLDYHDSNVKN |
| **P3** | HA2-17 | 20 | LDYHDSNVKNLYEKVRNQLK |
| **P3** | HA2-18 | 20 | LYEKVRNQLKNNAKEIGNGC |
| **P4** | HA2-19 | 20 | NNAKEIGNGCFEFYHKCDNT |
| **P4** | HA2-20 | 20 | FEFYHKCDNTCMESVKNGTY |
| **P4** | HA2-21 | 20 | CMESVKNGTYDYPKYSEEAK |
| **P4** | HA2-22 | 20 | DYPKYSEEAKLNREKIDGVK |
| **P4** | HA2-23 | 20 | LNREKIDGVKLESTRIYQIL |
| **P4** | HA2-24 | 20 | LESTRIYQILAIYSTVASSL |
| **P4** | HA2-25 | 20 | AIYSTVASSLVLVVSLGAIS |
| **P4** | HA2-26.2 | 15 | LGAISFWMCSNGSLQ |
| **P4** | HA2-27 | 15 | FWMCSNGSLQCRICI |

**TABLE S2. NP Peptide Pool (NP A/Michigan/45/2015)**

| **Pool (P1-P5)** | **Peptide #** | **Length** | **Amino Acid Sequence** |
| --- | --- | --- | --- |
| **P1** | NP-1 | 20 | MASQGTKRSYEQMETGGERQ |
| **P1** | NP-2 | 20 | EQMETGGERQDTTEIRASVG |
| **P1** | NP-3 | 20 | DTTEIRASVGRMIGGIGRFY |
| **P1** | NP-4 | 20 | RMIGGIGRFYIQMCTELKLS |
| **P1** | NP-5 | 20 | IQMCTELKLSDYDGRLIQNS |
| **P1** | NP-6 | 20 | DYDGRLIQNSITIERMVLSA |
| **P1** | NP-7 | 20 | ITIERMVLSAFDERRNKYLE |
| **P1** | NP-8 | 20 | FDERRNKYLEEHPSAGKDPK |
| **P1** | NP-9 | 20 | EHPSAGKDPKKTGGPIYRRI |
| **P1** | NP-10 | 20 | KTGGPIYRRIDGKWTRELIL |
| **P2** | NP-11 | 20 | DGKWTRELILYDKEEIRRVW |
| **P2** | NP-12 | 20 | YDKEEIRRVWRQANNGEDAT |
| **P2** | NP-13 | 20 | RQANNGEDATAGLTHIMIWH |
| **P2** | NP-14 | 20 | AGLTHIMIWHSNLNDATYQR |
| **P2** | NP-15 | 20 | SNLNDATYQRTRALVRTGMD |
| **P2** | NP-16 | 20 | TRALVRTGMDPRMCSLMQGS |
| **P2** | NP-17 | 20 | PRMCSLMQGSTLPRRSGAAG |
| **P2** | NP-18 | 20 | TLPRRSGAAGAAVKGVGTIA |
| **P2** | NP-19 | 20 | AAVKGVGTIAMELIRMIKRG |
| **P2** | NP-20 | 20 | MELIRMIKRGINDRNFWRGE |
| **P3** | NP-21 | 20 | INDRNFWRGENGRRTRVAYE |
| **P3** | NP-22 | 20 | NGRRTRVAYERMCNILKGKF |
| **P3** | NP-23 | 20 | RMCNILKGKFQTAAQRAMMD |
| **P3** | NP-24 | 20 | QTAAQRAMMDQVRESRNPGN |
| **P3** | NP-25 | 20 | QVRESRNPGNAEIEDLIFLA |
| **P3** | NP-26 | 20 | AEIEDLIFLARSALILRGSV |
| **P3** | NP-27 | 20 | RSALILRGSVAHKSCLPACV |
| **P3** | NP-28 | 20 | AHKSCLPACVYGLAVASGHD |
| **P3** | NP-29 | 20 | YGLAVASGHDFEREGYSLVG |
| **P3** | NP-30 | 20 | FEREGYSLVGIDPFKLLQNS |
| **P4** | NP-31 | 20 | IDPFKLLQNSQVVSLMRPNE |
| **P4** | NP-32 | 20 | QVVSLMRPNENPAHKSQLVW |
| **P4** | NP-33 | 20 | NPAHKSQLVWMACHSAAFED |
| **P4** | NP-34 | 20 | MACHSAAFEDLRVSSFIRGK |
| **P4** | NP-35 | 20 | LRVSSFIRGKKVIPRGKLST |
| **P4** | NP-36 | 20 | KVIPRGKLSTRGVQIASNEN |
| **P4** | NP-37 | 20 | RGVQIASNENVETMDSNTLE |
| **P4** | NP-38 | 20 | VETMDSNTLELRSRYWAIRT |
| **P4** | NP-39 | 20 | LRSRYWAIRTRSGGNTNQQK |
| **P4** | NP-40 | 20 | RSGGNTNQQKASAGQISVQP |
| **P5** | NP-41 | 20 | ASAGQISVQPTFSVQRNLPF |
| **P5** | NP-42 | 20 | TFSVQRNLPFERATVMAAFS |
| **P5** | NP-43 | 20 | ERATVMAAFSGNNEGRTSDM |
| **P5** | NP-44 | 20 | GNNEGRTSDMRTEVIRMMES |
| **P5** | NP-45 | 20 | RTEVIRMMESAKPEDLSFQG |
| **P5** | NP-46 | 20 | AKPEDLSFQGRGVFELSDEK |
| **P5** | NP-47 | 20 | RGVFELSDEKATNPIVPSFD |
| **P5** | NP-48 | 20 | ATNPIVPSFDMSNEGSYFFG |
| **P5** | NP-49 | 19 | MSNEGSYFFGDNAEEYDN |

**TABLE S3. Modified CTL Peptide Pool**

| **Pool** | **Peptide #** | **Length** | **Amino Acid Sequence** |
| --- | --- | --- | --- |
| EBV/CMV | CTL3 | 9 | GLCTLVAML |
| EBV/CMV | CTL5 | 9 | NLVPMVATV |
| EBV/CMV | CTL7 | 9 | RVRAYTYSK |
| EBV/CMV | CTL8 | 9 | RLRAEAQVK |
| EBV/CMV | CTL9 | 9 | IVTDFSVIK |
| EBV/CMV | CTL10 | 9 | ATIGTAMYK |
| EBV/CMV | CTL11 | 10 | DYCNVLNKEF |
| EBV/CMV | CTL13 | 9 | RPPIFIRRL |
| EBV/CMV | CTL14 | 10 | TPRVTGGGAM |
| EBV/CMV | CTL15 | 9 | QAKWRLQTL |
| EBV/CMV | CTL16 | 9 | FLRGRAYGL |
| EBV/CMV | CTL17 | 8 | RAKFKQLL |
| EBV/CMV | CTL20 | 9 | RRIYDLIEL |
| EBV/CMV | CTL21 | 9 | YPLHEQHGM |
| EBV/CMV | CTL22 | 10 | EENLLDFVRF |
| EBV/CMV | CTL23 | 10 | EFFWDANDIY |
| EBV/CMV | CTL25 | 9 | CLGGLLTMV |
| EBV/CMV | CTL28 | 10 | AVFDRKSDAK |
| EBV/CMV | CTL29 | 9 | TTVYPPSSTAK |
| EBV/CMV | CTL31 | 12 | SDEEEAIVAYTL |
| EBV/CMV | CTL33 | 9 | IPSINVHHY |
